# Supplementary material for: Content-rich biological network constructed by mining PubMed abstracts
Source: BMC Bioinformatics. 2004 Oct 8;5:147. doi: 10.1186/1471-2105-5-147 (PMC528731; doi:10.1186/1471-2105-5-147)
Supplement: Additional File 2 — The original results of the above study (non-essential files are deleted to keep the file size under the limit set by BMC bioinformatics). [file 1471-2105-5-147-S2.bz2 › chilibotAdditionalFile2/dip50/577ID9204896E10945/html/ANGPT2_TEK.html]

 


 **ANGPT2** and **TEK** 
  
Found 131 abstracts in PubMed, retrieved 50.  
 

 What does Google say? 
 PDF only 
| .edu only 

---

**Interactive relationship** (e.g. stimulation, inhibition, etc)

**Neutral relationship**- These data provide comprehensive evidence that VEGFR 1 and 2, and angiopoietin 1, angiopoietin 2  [ **ANGPT2** ]  and Tie 2  [ **TEK** ]  interactions may be involved in the preparation of endometrium for implantation, remodelling of the maternal vasculature and trophoblast invasion during the peri implantation period in this primate species.  Ref: 12887279 Reproduction, 2003
- Recent studies have implicated the Tie2  [ **TEK** ]  tyrosine kinase receptor and its main ligands angiopoietin 1 Ang 1 and angiopoietin 2  [ **ANGPT2** ]  Ang 2  [ **ANGPT2** ]  as crucial regulators of mural cell recruitment during angiogenesis.  Ref: 12876214 J Cell SciJ Cell Sci, 2003
- CONCLUSIONS VEGF and Ang2  [ **ANGPT2** ]  Tie2  [ **TEK** ]  interactions may play an important role in the pathogenesis of ROP.  Ref: 12815197 Ophthalmic Res, 2003
- Angiopoietin 1 Ang 1 promotes vascular maturation via the Tie 2  [ **TEK** ]  receptor, while angiopoietin 2  [ **ANGPT2** ]  Ang 2  [ **ANGPT2** ]  is its natural antagonist that destabilizes vessels and initiates neovascularization in the presence of vascular endothelial growth factor.  Ref: 11891175 Am J Pathol, 2002
- The Tie2  [ **TEK** ]  receptor protein showed the same pattern as Ang 2  [ **ANGPT2** ] .  Ref: 12653688 Clin Sci Lond , 2003
- Genetic rescue with Angiopoietin 1 corrects the lymphatic, but not the angiogenesis, defects, suggesting that Angiopoietin 2  [ **ANGPT2** ]  acts as a Tie2  [ **TEK** ]  agonist in the former setting, but as an antagonist in the latter setting.  Ref: 12361603 Dev Cell, 2002
- Angiopoietin 1 and angiopoietin 2  [ **ANGPT2** ]  share the same binding domains in the Tie 2  [ **TEK** ]  receptor involving the first Ig like loop and the epidermal growth factor like repeats.  Ref: 12427764 J Biol Chem, 2003
- The change in capillary density paralleled with VEGF and Ang 2  [ **ANGPT2** ]  expression, but was inversely related to Tie2  [ **TEK** ]  phosphorylation.  Ref: 12649074 Am J Physiol Heart Circ Physiol< MedlineTA>Am J Physiol Heart Circ Physiol, 2003
- These data suggest that it is endothelial induction of Ang 2  [ **ANGPT2** ]  in tumours that regulates vessel stability and supports targeting Tie2  [ **TEK** ]  as an effective novel anti angiogenic therapy in clear cell RCCs.  Ref: 12434420 J Pathol, 2002
- Ang1 and Ang2  [ **ANGPT2** ]  bind with similar affinity to the endothelial cell tyrosine kinase receptor Tie2  [ **TEK** ] .  Ref: 12138242 Oncology, 2002
- Angiopoietin 2  [ **ANGPT2** ]  Ang2  [ **ANGPT2** ]  appears to be a naturally occurring antagonist of the endothelial receptor tyrosine kinase Tie2  [ **TEK** ] , an important regulator of vascular stability.  Ref: 12692304 Proc Natl Acad Sci U S A, 2003
- It has been proposed that angiopoietins 1 Ang1 and 2 Ang2  [ **ANGPT2** ]  are pro and anti angiogenic owing to their respective agonist and antagonist signaling action through the Tie2  [ **TEK** ]  receptor.  Ref: 12163646 Proc Natl Acad Sci U S A, 2002
- Angiopoietin 1 Ang 1 and angiopoietin 2  [ **ANGPT2** ]  Ang 2  [ **ANGPT2** ]  have been identified as ligands with different effector functions of the vascular assembly and maturation mediating receptor tyrosine kinase Tie 2  [ **TEK** ] .  Ref: 12427764 J Biol Chem, 2003
- Hypoxia induced expression of NERF2 and Tie2  [ **TEK** ]  was blocked by angiopoietin 2  [ **ANGPT2** ] , a competitive inhibitor of angiopoietin 1, and by recombinant soluble extracellular domain of Tie2  [ **TEK** ]  but not by VEGF neutralizing antibodies.  Ref: 11967990 J Cell Biochem, 2002
- Thrombin induced secreted Ang 2  [ **ANGPT2** ]  inhibited the binding of recombinant 35 S Ang 1 to its Tie 2  [ **TEK** ]  Fc receptor, demonstrating functionality.  Ref: 11861279 Blood, 2002
- These findings identify an inhibitory role of Ang1 Tie2  [ **TEK** ]  receptor mediated vessel maturation in SCC growth and suggest that up regulation of its antagonist, Ang2  [ **ANGPT2** ] , during early stage epithelial tumorigenesis contributes to the angiogenic switch by counteracting specific vessel stabilizing effects of Ang1.  Ref: 11943723 Am J Pathol, 2002
- Using a combination of immunohistochemistry, laser capture microdissection and real time quantitative reverse transcription PCR, we showed that tumor derived VEGF significantly up regulated the expression of Ang 2  [ **ANGPT2** ]  in host stroma endothelial cells, resulting in markedly increased Ang 2  [ **ANGPT2** ]  Tie 2  [ **TEK** ]  mRNA copy number ratio in vivo.  Ref: 12810677 Cancer Res, 2003

**Non-interactive relationship** (e.g. studied together, co-existance, homology, etc.)

- To test the hypothesis that menorrhagia arises as a result of poor signal for vascular maturation, we have examined the expression of Ang 1, Ang 2  [ **ANGPT2** ] , and Tie 2  [ **TEK** ]  in endometrium throughout the menstrual cycle from 30 normal women and 28 patients with menorrhagia.  Ref: 11891175 Am J Pathol, 2002
- Moreover, Ang2  [ **ANGPT2** ]  gene expression showed no significant correlation with the Tie2  [ **TEK** ]  gene expression either in normal ovary with CL or in ovarian cancer.  Ref: 12138242 Oncology, 2002
- Generally Ang 1, Ang 2  [ **ANGPT2** ] , Tie1 and Tie2  [ **TEK** ]  mRNA levels were higher in RA synovial tissue compared to normal and OA synovial tissues, and RA synovial tissue fibroblasts.  Ref: 12010571 Arthritis Res, 2002
- At different stages of tumor growth, the histological aspects were described and sections were immunostained for VEGF, Ang 2  [ **ANGPT2** ]  and their receptors VEGFR 1, VEGFR 2 and Tie 2  [ **TEK** ] .  Ref: 12174896 Anticancer Res< MedlineTA>Anticancer Res, 2002
- Tie2  [ **TEK** ]  gene expression was positively correlated with Ang 2  [ **ANGPT2** ]  expression in cancers p = 0.001 and showed a borderline significant association with Ang 1 p = 0.06, but there was no significant relationship between Ang 1 and Ang 2  [ **ANGPT2** ]  p = 0.69.  Ref: 12434420 J Pathol, 2002
- Using RT PCR and immunohistochemistry we demonstrate that testicular vascular endothelial growth factor A VEGF A, ang 1, ang 2  [ **ANGPT2** ]  and the tie 2  [ **TEK** ]  angiopoietin receptor are expressed in the testis.  Ref: 12773423 Biol Reprod< MedlineTA>Biol Reprod, 2002
- In conclusion, Ang2  [ **ANGPT2** ]  and Tie2  [ **TEK** ]  expressions increased after ischemia reperfusion in the rat ventricular myocardium, while Ang1 and Tie1 expressions did not.  Ref: 12737621 Clin Sci Lond < MedlineTA>Clin Sci Lond , 2002
- The level of Ang 1 and Tie 2  [ **TEK** ]  mRNAs, but not that of Ang 2  [ **ANGPT2** ]  mRNA gradually decreased during a 14 d incubation of cord blood CD34 cells in a liquid culture.  Ref: 12605658 Eur J Haematol, 2003
- To test this hypothesis we measured capillary density, and the expressions of VEGF, angiopoietin 1 and 2 Ang 1 and Ang 2  [ **ANGPT2** ] , respectively and the Tie 2  [ **TEK** ]  receptor and its phosphorylation state pY Tie2  [ **TEK** ]  during repetitive episodes of myocardial ischemia in chronically instrumented canines.  Ref: 12649074 Am J Physiol Heart Circ Physiol< MedlineTA>Am J Physiol Heart Circ Physiol, 2003
- To explore the relationship of angiogenesis related angiopoietin 2  [ **ANGPT2** ]  gene and its receptor Tie2  [ **TEK** ]  with angiogenesis and the biology of hepatocellular carcinoma HCC, angiopoietin 2  [ **ANGPT2** ]  gene, Tie2  [ **TEK** ]  and CD34 protein expression in 22 resected HCC, 8 cirrhotic and 8 control liver specimens were investigated by in situ hybridization and immunohistochemistry respectively, and the level of angiopoietin 2  [ **ANGPT2** ]  and Tie2  [ **TEK** ]  expression in HCC were compared in terms of tumor biological parameters.  Ref: 12539584 J Tongji Med Univ, 2001
- While Angiopoietin 1 obligately activates its Tie2  [ **TEK** ]  receptor, Angiopoietin 2  [ **ANGPT2** ]  can activate Tie2  [ **TEK** ]  on some cells, while it blocks Tie2  [ **TEK** ]  activation on others.  Ref: 12361603 Dev Cell, 2002
- Tie 2  [ **TEK** ]  messenger RNA, the receptor of Ang 1 and Ang 2  [ **ANGPT2** ] , was detected at the same level in FNH as in normal liver.  Ref: 12612904 Gastroenterology, 2003
- Enzyme linked immunosorbent assay based competition assays and co immunoprecipitation experiments analyzing the binding of Ang 1 and Ang 2  [ **ANGPT2** ]  to truncation mutants of the extracellular domain of Tie 2  [ **TEK** ]  showed that the first Ig like loop of Tie 2  [ **TEK** ]  in combination with the epidermal growth factor EGF like repeats amino acids 1 360 is required for angiopoietin binding.  Ref: 12427764 J Biol Chem, 2003
- To determine the significance and regulation of the angiopoietin Ang pathway in highly vascular human renal cell carcinomas RCCs, this study has investigated the expression of the Ang 1, Ang 2  [ **ANGPT2** ] , Ang 4, and Tie2  [ **TEK** ]  genes in a series of normal n = 26 and neoplastic n = 45.  Ref: 12434420 J Pathol, 2002
- Ang 1, Ang 2  [ **ANGPT2** ] , Tie1 and Tie2  [ **TEK** ]  mRNA and protein expression were quantified in synovial tissues and RA synovial tissue fibroblasts with real time reverse transcription polymerase chain reaction and western blot analysis.In RA, Ang 1 positive immunostaining on lining cells, macrophages and endothelial cells was significantly higher than in OA and normal synovial tissue.  Ref: 12010571 Arthritis Res, 2002
- To understand the molecular interactions of the angiopoietins with their receptor, we have studied the binding of Ang 1 and Ang 2  [ **ANGPT2** ]  to the Tie 2  [ **TEK** ]  receptor.  Ref: 12427764 J Biol Chem, 2003
- Immunohistochemical analysis was used to determine the expression of Ang 1, Ang 2  [ **ANGPT2** ] , Tie1 and Tie2  [ **TEK** ]  in synovial tissue of normal subjects and those with RA and OA.  Ref: 12010571 Arthritis Res, 2002
- Thus cyclical mechanical stretch activates the expression of angiopoietin 2  [ **ANGPT2** ]  and the Tie2  [ **TEK** ]  receptor, but not angiopoietin 1 or the Tie1 receptor, in cultured HUVECs.  Ref: 12653688 Clin Sci Lond , 2003
- RESULTS The Ang1, Ang2  [ **ANGPT2** ] , and Tie2  [ **TEK** ]  gene expression in normal ovary with CL ranged from 0.18 to 1.06 median 0.5.31 2.64 median 1.01, and 0.10 0.47 median 0.20, respectively.  Ref: 12138242 Oncology, 2002
- RESULTS In human epiretinal membranes surgically obtained from eyes with ischemic retinal disorders, substantial upregulation of angiopoietin 2  [ **ANGPT2** ]  Ang2  [ **ANGPT2** ]  and the receptor Tie2  [ **TEK** ]  was recorded than in those from eyes with nonischemic diseases.  Ref: 12506101 Invest Ophthalmol Vis Sci, 2003
- These included familiar endothelial cell associated genes such as VEGF, VEGFR 1, VEGFR 2, angiopoietin 2  [ **ANGPT2** ] , Tie1, Tie2  [ **TEK** ] , Edg1 receptor, VE cadherin, claudin 5, connexin 37, CD31, and CD34.  Ref: 12842817 Am J Physiol Heart Circ Physiol< MedlineTA>Am J Physiol Heart Circ Physiol, 2003
- Cyclical mechanical stretch increased and Gö6976 or herbimycin A abrogated these increases the immunohistochemical labelling of angiopoietin 2  [ **ANGPT2** ]  and Tie2  [ **TEK** ]  receptor after a 6 h stretch.  Ref: 12653688 Clin Sci Lond , 2003
- Western blot analyses showed that HBO enhanced the expression of angiopoietin 2  [ **ANGPT2** ]  Ang2  [ **ANGPT2** ]  with no effect on the expression of Tie2  [ **TEK** ] , angiopoietin 1, and VEGF.  Ref: 12176040 Biochem Biophys Res Commun, 2002
- However, there was a significant increase in expression of ANG 2  [ **ANGPT2** ]  and its receptor Tie 2  [ **TEK** ]  in menorrhagic tissues, which may be important in destabilizing the endometrial vasculature.  Ref: 12477527 Fertil Steril, 2002
- In conclusion, our data suggest that both hypoxia and VEGF treatment differentially regulate the angiopoietin Tie2  [ **TEK** ]  system in the two vascular cells and that, particularly in BRP, the regulation of Ang1, but not Ang2  [ **ANGPT2** ] , and Tie2  [ **TEK** ]  expression may play an important role in vascular development.  Ref: 12686170 Microvasc Res, 2003
- EXPERIMENTAL DESIGN We investigated immunohistochemically the expression patterns and levels of antiangiogenic factor and its receptor, thrombospondin 1 TSP 1 and CD36, and four angiogenic factors, vascular endothelial growth factor VEGF, VEGF C, angiopoietin 2  [ **ANGPT2** ]  Ang 2  [ **ANGPT2** ] , and Tie 2  [ **TEK** ] , in the primary tumors of 75 papillary thyroid carcinoma patients.  Ref: 12006528 Clin Cancer Res, 2002
- RESULTS Ang 2  [ **ANGPT2** ]  and Tie 2  [ **TEK** ]  were detected in the endothelial cells of vessels surrounded by tumor cells, occuring early in our study, with immunostaining taking place from day 4 to day 24.  Ref: 12174896 Anticancer Res< MedlineTA>Anticancer Res, 2002
- However, there was significantly greater Ang 1 p = 0.02, Ang 2  [ **ANGPT2** ]  p = 0.03, and Tie2  [ **TEK** ]  p = 0.04 mRNA abundance in clear cell than in chromophil RCCs.  Ref: 12434420 J Pathol, 2002
- Expression of angiopoietin 2  [ **ANGPT2** ]  gene and its receptor Tie2  [ **TEK** ]  in hepatocellular carcinoma.  Ref: 12539584 J Tongji Med Univ, 2001
- Furthermore, cellular distribution of Ang1 and Ang2  [ **ANGPT2** ]  mRNA was examined by in situ hybridization, and localization of Tie2  [ **TEK** ]  was studied by immunochemistry.  Ref: 12138242 Oncology, 2002
- METHODS The mRNA levels of VEGF, Ang 1, Ang 2  [ **ANGPT2** ] , Tie 1, and Tie 2  [ **TEK** ]  were measured by semiquantitative reverse transcription polymerase chain reaction RT PCR.  Ref: 11978190 Acta Pharmacol Sin, 2002
- The gene expression levels of Ang2  [ **ANGPT2** ]  and Tie2  [ **TEK** ]  were statistically the same in both groups.  Ref: 12138242 Oncology, 2002
- Polyclonal antibodies specific for Ang1, Ang2  [ **ANGPT2** ] , Tie2  [ **TEK** ]  and VEGF were used for immunostaining.  Ref: 12815197 Ophthalmic Res, 2003
- MATERIAL AND METHODS We have measured the expression of angiogenesis regulating genes angiopoietin 1 Ang 1, angiopoietin 1 Ang 2  [ **ANGPT2** ]  and their receptor Tie 2  [ **TEK** ] , vascular endothelial growth factor VEGF and its receptors VEGFR 1 and VEGFR 2 in sorted population of CD34 and CD34 CD133 cells from human cord blood and bone marrow, and in their differentiating progeny, using real time reverse transcriptase polymerase chain reaction.  Ref: 12605658 Eur J Haematol, 2003
- The study of angiopoietin 1 Ang 1 and angiopoietin 2  [ **ANGPT2** ]  Ang 2  [ **ANGPT2** ] , ligands of receptor tyrosine kinase 2 Tie 2  [ **TEK** ] , showed that while stroma derived Ang 2  [ **ANGPT2** ]  was increased, epidermal Ang 1 expression was completely abolished at early papilloma formation.  Ref: 12766907 Mol Carcinog, 2003
- Cyclical mechanical stretch enhances angiopoietin 2  [ **ANGPT2** ]  and Tie2  [ **TEK** ]  receptor expression in cultured human umbilical vein endothelial cells.  Ref: 12653688 Clin Sci Lond , 2003
- Colocalization of tie2  [ **TEK** ] , angiopoietin 2  [ **ANGPT2** ]  and vascular endothelial growth factor in fibrovascular membrane from patients with retinopathy of prematurity.  Ref: 12815197 Ophthalmic Res, 2003
- AIM To study the expressions of vascular endothelial growth factor VEG F, angiopoietin 1 Ang 1, angiopoietin 2  [ **ANGPT2** ]  Ang 2  [ **ANGPT2** ] , Tie 1, and Tie 2  [ **TEK** ]  in C57BL 6 mouse brain after permanent focal cerebral ischemia.  Ref: 11978190 Acta Pharmacol Sin, 2002
- A new study in this issue of Developmental Cell shows that Angiopoietin 2  [ **ANGPT2** ]  Ang2  [ **ANGPT2** ] , a ligand for the endothelial Tie2  [ **TEK** ]  receptor tyrosine kinase, has a dual function in the processes of postnatal angiogenesis and vascular remodeling.  Ref: 12361591 Dev Cell, 2002
- Immunohistochemical study also showed increased immunoreactivity of Ang2  [ **ANGPT2** ]  and Tie2  [ **TEK** ]  in the jeopardized myocardium after ischemia reperfusion.  Ref: 12737621 Clin Sci Lond < MedlineTA>Clin Sci Lond , 2002
- In addition, they strongly expressed angiopoietin 1 Ang 1 but not angiopoietin 2  [ **ANGPT2** ] , Tie 1, or Tie 2  [ **TEK** ] .  Ref: 11950897 Lab Invest, 2002
- METHODS Ang1, Ang2  [ **ANGPT2** ] , and Tie2  [ **TEK** ]  gene expression in 14 normal ovaries with corpus luteum CL and in 19 cases of ovarian cancer were analyzed by polymerase chain reaction of RNA after reverse transcription.  Ref: 12138242 Oncology, 2002
- angiopoietin 2  [ **ANGPT2** ]  Tie2  [ **TEK** ]  expression in human hepatic carcinoma is closely related with angiogenesis, which are probably involved in the HCC angiogenesis regulation, promoting the development and metastasis of human hepatic cancer.  Ref: 12539584 J Tongji Med Univ, 2001
- Specifically, 1 VEGF Flt and Ang 1 Tie 2  [ **TEK** ]  may promote trophoblast growth, 2 VEGF KDR and Ang 1 Tie 2  [ **TEK** ]  may support fetoplacental vascular development and stabilization, 3 sFlt may balance VEGF actions, and 4 Ang 2  [ **ANGPT2** ]  Tie 2  [ **TEK** ]  may remodel the maternal vasculature.  Ref: 11870089 Biol Reprod, 2002
- Immunoprecipitation showed phosphorylation of MPC Tie2  [ **TEK** ]  on tyrosine residues upon stimulation with Ang 1 or Ang 2  [ **ANGPT2** ] .  Ref: 12876214 J Cell SciJ Cell Sci, 2002
- To investigate the specific role of Ang2  [ **ANGPT2** ]  in the adult vasculature, we generated a nuclease resistant RNA aptamer that binds and inhibits Ang2  [ **ANGPT2** ]  but not the related Tie2  [ **TEK** ]  agonist, angiopoietin 1.  Ref: 12692304 Proc Natl Acad Sci U S A, 2003
- RESULTS A higher expression of Ang 1, Ang 2  [ **ANGPT2** ] , and Tie 2  [ **TEK** ]  mRNAs was detected in CD34 CD133 cord blood cells as compared with CD34 CD133 fraction, but no expression of these genes was detected in burst forming unit erythroid BFU E nor colony forming unit granulocyte macrophage CFU GM colonies.  Ref: 12605658 Eur J Haematol, 2003
- Using an ovine model of PI IUGR we have observed changes in the expression of vascular endothelial growth factor, placental growth factor, their common receptors, as well as angiopoietin 2  [ **ANGPT2** ]  and its receptor, Tie 2  [ **TEK** ] .  Ref: 11978069 Placenta, 2002
- Ang2  [ **ANGPT2** ]  stained positively in 18 of 38 47.3% serial sections where Tie2  [ **TEK** ]  was present, and was also co expressed with VEGF and factor VIII.  Ref: 12815197 Ophthalmic Res, 2003
- Ang2  [ **ANGPT2** ]  and Tie2  [ **TEK** ]  immunoreactivity was induced mainly in capillaries and postcapillary venules in chronic allografts when compared with syngeneic controls, but no immunoreactivity was found in arterial endothelium.  Ref: 12628953 Circulation, 2003
- No significant relationships were observed in clear cell carcinomas between Ang 1, Ang 2  [ **ANGPT2** ]  and Tie2  [ **TEK** ]  mRNA abundance and patient sex, patient age, or tumour size p > 0.05.  Ref: 12434420 J Pathol, 2002
- After middle cerebral artery occlusion MCAO, the expressions of VEGF, Ang 2  [ **ANGPT2** ] , and Tie 2  [ **TEK** ]  mRNA were dramatically increased in the infarcted cortex and the elevation was remained through 7 d of ischemia.  Ref: 11978190 Acta Pharmacol Sin, 2002
- Here, we studied the roles of Ang1, its natural antagonist Ang2  [ **ANGPT2** ] , and their receptor Tie2  [ **TEK** ]  in rat cardiac allograft arteriosclerosis.  Ref: 12628953 Circulation, 2003
- Angiopoetin 1 Ang1, angiopoietin 2  [ **ANGPT2** ]  Ang2  [ **ANGPT2** ] , Tie 1, and Tie 2  [ **TEK** ]  were measured immediately after relief of occlusion, 60 min, 4, and 72 h after reperfusion by Northern blot, Western blot, and immunohistochemical staining.  Ref: 12737621 Clin Sci Lond < MedlineTA>Clin Sci Lond , 2003
- mRNAs for VEGF, VEGFR 1 and VEGFR 2, angiopoietin 1, angiopoietin 2  [ **ANGPT2** ]  and their receptor Tie 2  [ **TEK** ]  were localized and quantified by in situ hybridization.  Ref: 12887279 Reproduction, 2003
- Similar to protein expression, the levels of angiopoietin 2  [ **ANGPT2** ]  and Tie2  [ **TEK** ]  receptor mRNAs in HUVECs increased 3.1 fold and 2.5 fold respectively after stretch for 6 h.  Ref: 12653688 Clin Sci Lond , 2003
- clear cell n = 35, papillary n = 10 human kidney tissues, examined the pattern of Ang 2  [ **ANGPT2** ]  and Tie2  [ **TEK** ]  protein expression, and correlated expression with clinicopathological variables.  Ref: 12434420 J Pathol, 2002
- Conversely, the first 360 amino acids Ig like domain plus EGF like repeats of the Tie 2  [ **TEK** ]  receptor are necessary and sufficient to bind both Ang 1 and Ang 2  [ **ANGPT2** ] , which suggests that differential receptor binding is not likely to be responsible for the different functions of Ang 1 and Ang 2  [ **ANGPT2** ] .  Ref: 12427764 J Biol Chem, 2003
- Expression of angiopoietin 1, angiopoietin 2  [ **ANGPT2** ] , and Tie2  [ **TEK** ]  genes in normal ovary with corpus luteum and in ovarian cancer.  Ref: 12138242 Oncology, 2002
- RESULTS Low mRNA levels of VEGF, Ang 1, Ang 2  [ **ANGPT2** ] , Tie 1, and Tie 2  [ **TEK** ]  were constitutively expressed in the normal cortex of mouse.  Ref: 11978190 Acta Pharmacol Sin, 2002
- CONCLUSION S Differences in the expression of ANG 2  [ **ANGPT2** ] , Tie 2  [ **TEK** ] , Tie 1, and eNOS were found in menorrhagic endometrium, which may represent a new target for therapeutic intervention to correct menorrhagic conditions.  Ref: 12477527 Fertil Steril, 2002
- The distinct roles of angiopoietin Ang 1 and Ang2  [ **ANGPT2** ] , counteracting ligands for the endothelium specific Tie2  [ **TEK** ]  receptor, in tumor development and progression have remained poorly understood.  Ref: 11943723 Am J Pathol, 2002
- These increases in angiopoietin 2  [ **ANGPT2** ]  and Tie2  [ **TEK** ]  receptor proteins at 6 h were blocked by the addition 30 min before stretch of the protein kinase C inhibitor Gö6976 16 nM or the tyrosine kinase inhibitor herbimycin A 24 microM.  Ref: 12653688 Clin Sci Lond , 2003
- ... Ang 2  [ **ANGPT2** ]  and Ang 3 inhibit Ang 1 induced Tie2  [ **TEK** ]  phosphorylation.  Ref: 12783598 Expert Opin Investig Drugs, 2003
- Ang 1, Ang 2  [ **ANGPT2** ]  and Tie2  [ **TEK** ] , but not Ang 4 mRNA, were detected in normal and tumour samples.  Ref: 12434420 J Pathol, 2002
- CONCLUSIONS A specific increase of Ang 1 Ang 2  [ **ANGPT2** ]  ratio in FNH, in the presence of the functional Tie 2  [ **TEK** ]  receptor, might be involved in the formation of hyperplastic and dystrophic vessels of FNH.  Ref: 12612904 Gastroenterology, 2003
- In this study, we investigated the expression of Ang 1, Ang 2  [ **ANGPT2** ] , Tie 2  [ **TEK** ] , and vascular endothelial growth factor VEGF in surgically resected specimens from 46 patients with HCC to determine their potential role in tumor angiogenesis and its progression.  Ref: 12717391 Hepatology, 2003
- However, using bovine aortic endothelial cells BAEC, we found that only Ang2  [ **ANGPT2** ]  expression, but neither Ang1 nor Tie2  [ **TEK** ] , responded to these two angiogenic stimuli, which was consistent with many previous reports.  Ref: 12686170 Microvasc Res, 2003
- Angiopoietins are a recently discovered family of angiogenic factors that interact with the endothelial receptor tyrosine kinase Tie2  [ **TEK** ] , either as agonists angiopoietin 1 or as context dependent agonists antagonists angiopoietin 2  [ **ANGPT2** ] .  Ref: 12469114 Nat Struct Biol, 2003
- Increased expression of angiopoietin 2  [ **ANGPT2** ]  and Tie2  [ **TEK** ]  receptor in a rat model of myocardial ischemia reperfusion.  Ref: 12737621 Clin Sci Lond < MedlineTA>Clin Sci Lond , 2003
